# The Prevalence of *Aliarcobacter* Species in the Fecal Microbiota of Farm Animals and Potential Effective Agents for Their Treatment: A Review of the Past Decade

**DOI:** 10.3390/microorganisms10122430

**Published:** 2022-12-08

**Authors:** Cansu Çelik, Orhan Pınar, Nisa Sipahi

**Affiliations:** 1 Food Technology Program, Food Processing Department, Vocational School of Veterinary Medicine, Istanbul University-Cerrahpasa, 34320 Istanbul, Türkiye; 2 Equine and Equine Training Program, Vocational School of Veterinary Medicine, Istanbul University-Cerrahpasa, 34320 Istanbul, Türkiye; 3 Traditional and Complementary Medicine Applied and Research Centre, Duzce University, 81620 Duzce, Türkiye

**Keywords:** *Aliarcobacter*, livestock, fecal microbiota, antimicrobial resistance

## Abstract

There is an endless demand for livestock-originated food, so it is necessary to elucidate the hazard points for livestock breeding. Pathogens are one of the hazard points that threaten the biosecurity of farm-animal breeding and public health. As a potential foodborne pathogen, *Aliarcobacter* is a member of the intestinal microbiota of farm animals with and without diarrhea. *Aliarcobacter* spp. are capable of colonizing livestock intestines and are transmitted through the feces. Hence, they endanger slaughterhouses and milk products with fecal contamination. They also have other, rarer, vertical and horizontal transmission routes, including the offspring that abort in farm animals. Gastrointestinal symptoms and abort cases demonstrate potential financial losses to the industry. Viewed from this perspective, the global circulation of farm-animal products is a significant route for zoonotic agents, including *Aliarcobacter*. In the last decade, worldwide prevalence of *Aliarcobacter* in fecal samples has ranged from 0.8% in Italy to 100% in Turkey. Furthermore, antibiotic resistance is recognized as a new type of environmental pollutant and has become a hot topic in animal breeding and the food industry. Increasing antibiotic resistance has become a significant problem impacting productivity. The increase in antimicrobial resistance rates in *Aliarcobacter* is caused by the misuse of antimicrobial drugs in livestock animals, leading to the acquiring of resistance genes from other bacteria, as well as mutations in current resistance genes. The most resistant strains are *A. butzleri*, *A. cryaerophilus*, and *A. skirrowii*. This review analyzes recent findings from the past decade on the prevalence of *Aliarcobacter* in the intestinal microbiota and the current effective antibiotics against *Aliarcobacter*. The paper also highlights that *A. cryaerophilus* and *A. skirrowii* are found frequently in diarrheal feces, indicating that *Aliarcobacter* should be studied further in livestock diarrheal diseases. Moreover, *Aliarcobacter*-infected farm animals can be treated with only a limited number of antibiotics, such as enrofloxacin, doxycycline, oxytetracycline, and gentamicin.

## 1. Introduction

The genus *Arcobacter* belongs to the family Campylobactereaceae [1]. The number of novel species has increased dramatically in the last five years. The genus *Arcobacter* has been divided into six genera—*Arcobacter*, *Aliarcobacter* gen. nov., *Pseudoarcobacter* gen. nov., *Halarcobacter* gen. nov., *Malaciobacter* gen. nov., and *Poseidonibacter* gen. nov. The 16S rRNA genetic similarity of some species has been observed to be low. The genus *Aliarcobacter* gen. nov. comprises *Aliarcobacter cryaerophilus* comb. nov., *Aliarcobacter butzleri* comb. nov., *Aliarcobacter skirrowii* comb. nov., *Aliarcobacter thereius* comb. nov., *Aliarcobacter trophiarum* comb. nov., *Aliarcobacter lanthieri* comb. nov., and *Aliarcobacter faecis* comb. nov. [2,3]. While 29 species were identified before 2019 [4], this number was updated to 33 in 2020 [5]. *A. vitoriensis* sp. nov. [6] and *A. vandammei* sp. nov. [7] are recorded as the two most recently identified species. Furthermore, *A. faecis*, and *A. lanthieri* have been identified as emerging pathogens that could harm humans and animals [8].

The genus *Aliarcobacter*, with its new name, is a Gram-negative curved rod, and is motile with a single polar flagellum. It is 0.2 to 0.5 μm in diameter and 1 to 3 μm long, and is oxidase- and catalase-positive. *Aliarcobacter* do not produce fluorescent pigments. Some species may grow in the presence of safranin or oxgall, but they do not occur in the presence of 2, 3, 5-triphenyltetrazolium chloride (0.04%, *w*/*v*), glycine (1% *w*/*v*), or 4% NaCl [2]. Colonies on blood-agar plates after three days of incubation have a diameter of 2 to 4 mm, and most are round and whitish.

However, there is still no standard isolation protocol, so the current isolation techniques may not lead us to a reliable result [9]. Nevertheless, there have been different approaches for isolating *Aliarcobacter* in recent years. The study by Merga et al. [10] compared the five most commonly used isolation methods and found that the “*Arcobacter* broth-mCCDA-Columbia Agar” isolation method [11,12], in which the selectivity was achieved with selective antibiotics instead of filtration, was more specific and sensitive than the other methods. *Aliarcobacter* species were identified by Celik and Ikiz [13] using the multiplex polymerase chain reaction (mPCR) technique in all isolates, and this proved the selectivity of the “*Arcobacter* broth-mCCDA-Columbia Agar” method. Moreover, *A. skirrowii* was isolated at a high rate in Celik and Ikiz’s [13] investigation, which was similar to the results of Merga et al. [10]. This was surprising, as *A. skirrowii* strains were known to be the most susceptible strains to antibiotics [14]. It was reported that *A. skirrowii* was the species that showed the least sensitivity to the substances used in the medium, followed by *A. butzleri* and *A. cryaerophilus* [10].

*Aliarcobacter* has been isolated from cases with clinical symptoms, such as acute or chronic watery diarrhea (21%) in pigs and abortions (41.8%) in sows [15,16,17]. Nachamkin et al. [18] reported that *A. butzleri* was isolated from fecal samples of swine, cattle, horses, ostriches, and tortoises with diarrhea, and *A. skirrowii* was isolated from sheep and cattle with diarrhea and hemorrhagic colitis. Vandamme et al. [19,20,21] detected *A. skirrowii* and *A. butzleri* in lambs with enteritis. However, there have been many studies that have proven that *Aliarcobacter* strains can be detected in healthy cattle, sheep, and pigs [10,20,21]. Regarding healthy chicken fecal samples, the prevalence is low as a result of avian body temperature being 41.8 °C, being that the majority of strains grow at 18 °C to 37 °C [22,23]. To sum up, water, animal, human clinical specimens, foodstuffs, and food facilities are natural habitats and environments of *Aliarcobacter* [5]. 

The purposes of this review were to (i) show the prevalence rates of *Aliarcobacter* species in the feces of farm animals and the change in antibiotic-resistance rates from 2012 to 2022, as well as to (ii) indicate effective antibiotics for treatment and to (iii) describe the factors influencing prevalence.

Research information was focused on studies of the prevalence of *A. butzleri*, *A. cryaerophilus*, and *A. skirrowii* in the feces of farm animals over the past decade. A database was gathered from experiments where *Aliarcobacter* species were specified. This included publications that were obtained from the ISI Web of Science database, Scopus, and Springer Link using the words as keywords.

### 1.1. Virulence Factors

In the last 20 years, research on *Aliarcobacter* pathogenicity and virulence mechanisms has contributed to the fight against the disease [1,24]. Putative virulence determinants have been identified in *Campylobacter*. However, until recently, little was known about the genes that directly generate the infection of *Aliarcobacter*, although recent research has shed some light on virulence factors. 

*A. butzleri* has been described as potentially the most virulent *Aliarcobacter* species, and the fecal shedding was observed to be longer for *A. Butzleri* than for other *Aliarcobacter* species. Furthermore, *A. Butzleri* was detected to have spread to the majority of tissues, including the liver, kidney, ileum, and brain. On the contrary, *A. Cryaerophilus* and *A. Skirrowii* were not seen in these tissues, indicating that these agents may not pass through the intestinal wall [25]. 

The pathogenesis of *A. Butzleri* is dependent on the host species and breed. In contrast to the colonization and mortality found in Beltsville white turkeys, *A. Butzleri* was unable to colonize standard chickens and turkeys [26]. Rats that had been infected with *A. Butzleri* or *A. Cryaerophilus* strains developed diarrhea, electrolyte imbalance, and changes in haematological parameters. *Aliarcobacter* disease may be dose-dependent, indicating that oral doses resulted in varying the progress of disease between mild and diarrhea [27].

Villarruel-Lopez et al. [28] sampled beef, pork, and chicken meat to determine *Aliarcobacter* cytotoxicity against Vero cells. Only the pork meat was found to have *A. Butzleri*, *A. Skirrowii*, and *A. Cryaerophilus.* It was reported that 95% of the *Aliarcobacter* isolates produced a virulence mechanism against Vero cells, including cell elongation and the formation of vacuoles. It was the first time that *Aliarcobacter* spp. Were recorded as producing a vacuolating toxin. By using PCR, Miller et al. [29] detected that *A. Butzleri* ATCC 49616 had *cadF* and *cj1349* genes encoding fibronectin-binding proteins, which promote the binding of bacteria to intestinal cells; *ciaB*, which encodes invasion antigen B that contributes to host cell invasion; the virulence factor *mviN* (inner membrane protein required for peptidoglycan biosynthesis); the gene *pldA*, which encodes outer membrane phospholipase A associated with lysis of erythrocytes; the hemolysin gene *tlyA*; the iron-regulated outer membrane protein (*irgA*); filamentous hemagglutinin (*hecA*); and hemolysin-activation protein (the gene *hecB*).

There are other important factors, such as enterotoxins, adherence, invasiveness, and penetrability, that influence the pathogenesis of bacteria. *Aliarcobacter* was determined to be capable of generating disease by attaching to the surface of epithelial cells or invading the intestinal epithelial cells and replicating in the intestinal lumen [30]. Douidah et al. [31] designed a rapid detection method (PCR) for putative virulence genes for *Aliarcobacter* strains. *hecA* and *hecB* were detected significantly more in cattle strains than in pig and chicken strains (*p* < 0.05). *tlyA* was found more frequently in *A. cryaerophilus* strains from pigs than in those from chickens (*p* < 0.05).

Levican et al. [24] emphasized that *Aliarcobacter* species are potential pathogens of humans and also described *A. trophiarum* and *A. defluvii* as potentially more virulent. In their research, they isolated *Aliarcobacter* species mostly from animal feces and sewage and nearly all were found to be adhesive to Caco-2 cells. Among their isolates the most invasive strains were detected from *A. trophiarum* (3/3), *A. skirrowii* (1/2), *A. cryaerophilus* (1/5), *A. butzleri* (2/12), and *A. defluvii* (1/8). As a remarkable result, *A. trophiarum* (all from feces of pig and chicken) was significantly (*p* < 0.05) more invasive than the other species.

The authors continued to detect putative genes by PCR from different isolates in livestock. Sekhar et al. [32] studied fecal samples and predominantly detected the genes of *ciaB*, *cj1349*, *mviN*, *cadF*, *pldA*, and *tlyA*. In addition, the presence of cytolethal distending toxin (*cdtA*, *cdtB*, and *cdtC*) genes in *A. faecis* and *A. lanthieri* reference strains was revealed with high frequencies. *cadF*, *ciaB*, *mviN*, *pldA*, and *tylA* are considered common virulence genes in *A. butzleri* and *A. skirrowii* strains [33]. Although *A. thereius* and *A. mytili* lack virulence genes, they were able to bind and invade Caco-2 cell lines [24].

Potential virulence factors must be evaluated in order to determine their clinical significance in humans and animals. Based on the data so far, we understand that *Aliarcobacter* species are capable of attaching to and penetrating the host’s intestinal epithelial cells, causing inflammatory reactions, septicemia, and enteritis [31]. The presence of virulence genes and their cytopathogenic activity on in vitro cell lines led the International Commission on Microbiological Specifications for Foods to classify *A. butzleri* as a “serious hazard” to human health [34].

In brief, the way *Aliarcobacter* creates disease and its virulence factors have been better understood after the development of in vivo laboratory animal models, cell culture techniques, and rapid and accurate PCR assays. 

### 1.2. Livestock as a Reservoirs of Aliarcobacter

*Aliarcobacter* spp. may be found all over the world, with infectious sources including livestock and water [35,36,37]. The routes of transmission of *Aliarcobacter* among animals are still under investigation. In a study conducted in the Netherlands on pregnant pigs, it was investigated whether the mother could transmit the agent to her offspring through the intrauterine route, and *A. skirrowii* has been observed to be the most prominent *Aliarcobacter* species in intrauterine transmission. In addition, postpartum infections caused by *Aliarcobacter* in piglets were also investigated, and it was stated that the agent was transmitted to the offspring from the mother, other newborns, and the environment. Therefore, it is clear that *Aliarcobacter* can be transmitted to animals both vertically and horizontally [38]. *Aliarcobacter* spp. has been investigated many times in the stools of livestock animals in the last decade (Table 1) using various isolation methods and molecular techniques [10,13,20,39]. The results indicate that cattle and sheep are significant intestinal carriers of *Aliarcobacter* spp. [40]. Moreover, *Aliarcobacter* is found in pigs at all stages of production, from piglets through to ground-meat [41]. *A. butzleri*, *A. cryaerophilus*, and *A. skirrowii* have been related to animal diseases and have been isolated from milk samples from a mastitis-affected cow, aborted livestock fetuses, and diarrhea-affected cattle [42,43].

Moreover, water, domestic pets, migratory birds, farm equipment, transport vehicles, feed, and farmers may play an important role in spreading *Aliarcobacter* spp. [32,55,56]. Farm animals are accepted as a potential source of disease since they have higher prevalence rates of *Aliarcobacter* species [57]. There was no disease connection in chickens, ducks, turkeys, and domesticated geese. However, it was suggested that diverse poultry species could act as a natural reservoir for *Aliarcobacter* spp. [58,59]. The gut content of chickens was found to be the source of transmission of *Aliarcobacter* spp. into slaughterhouses. *Aliarcobacter* species from the intestine contaminate the environment [58]. Small ruminants endanger slaughterhouses and milk products with fecal contamination, so they pose possible sources of foodborne infections. 

There may be some cases of change in the recovery of *Aliarcobacter* isolates over time in the same animal. Temporary colonization of sheep, low diagnostic levels, or irregular excretion of *Aliarcobacter* in feces has shed light on this inconsistency [60]. Poultry products, as reservoirs for *Aliarcobacter*, may pose a hazard for public health, although they are incapable of colonizing the gut of chickens, as the internal temperature of chickens (40.5 to 42 °C) may not provide an optimal environment for *Aliarcobacter* species because their growth temperature range is between 26 and 30 °C. However, *Aliarcobacter* can be transmitted to consumers through poultry products due to the processing and storage of poultry meat below 4 °C and at room temperature. In humans, diarrheal illness-associated *Aliarcobacter* can be spread through drinking water and surface and ground water and, in addition, livestock animals and raw meat are considered a source of *Aliarcobacter* infection in humans. Although *A. butzleri* and *A. cryaerophilus* have been found in slaughter equipment, the mechanism of transmission of *Aliarcobacter* spp. to humans has not yet been clarified [61]. Infections with *A. butzleri* cause diarrhea and abdominal pain, as well as nausea, vomiting, and fever in humans, whereas *A. skirrowii* strains lead to diarrhea. *A. butzleri* shows similar symptoms to *Campylobacter jejuni*, but a noteworthy difference is that *Aliarcobacter* causes more persistent, watery, and less-bloody diarrhea [62].

### 1.3. Factors Affecting Aliarcobacter Prevalence

There are many factors that affect the colonization of *Aliarcobacter***,** such as animal age, the season of the sampling, geographical location, isolation method, sampling type, farm management, and symptoms of gastrointestinal disease [10,26]. Although Kabeya et al. [20] reported that season does not affect *Aliarcobacter* prevalence, many studies have found a positive or negative correlation between temperature and prevalence. It was observed that *Aliarcobacter* was present in almost all of the samples collected by Fisher et al. [63] in August, but during January or April, they observed *Aliarcobacter* species in a only a few samples. Similarly, the prevalence of *A. butzleri* was found to be higher in July (76.9%) and August (77.8%), compared to September (42.9%), in the study of Levican et al. [64], and Wesley et al. [65] detected *Aliarcobacter* more frequently in cattle fecal samples that were taken after the start of May (26.7%) than in those taken earlier (16.6%). Significantly, contrary to these findings, *A. butzleri* was detected more in samples collected during the winter–spring period (29%) than from those of the summer–autumn period (8%) [66]. Furthermore, an increase in the prevalence of *Aliarcobacter* strains in sheep was seen in autumn and in winter [13,67]. According to Grove-White et al. [67], the effect of variation in farm-management between dairy cattle and sheep was revealed, with *Aliarcobacter* spp. being isolated at a greater rate in ruminants raised in closed barns (50.1%) than in animals raised in pastures (20.9%); however the opposite was detected in *Camplylobacter* samples [68]. The reasons for this difference might be due to variations in the sources of bacterial fecal contamination, as well as the geographical and climatic features of farm sites [66].

Ho et al. [58] reported that the recovery of *Aliarcobacter* species depends on the sampling size and place. For instance, in chickens, aerotolerant *Aliarcobacter* species may prefer the ileum over the anaerobic caecum. It is also necessary to consider the difference in culturing methods and incubation conditions that can affect the prevalence and diversity of *Aliarcobacter* spp. [64,69]. Golla et al. [70] observed that there was a positive correlation between age and the prevalence of *Aliarcobacter*. In healthy cattle, 16% of rectal swab samples were found to be *A. butzleri*, whereas only 2% of those from healthy young cattle were identified as *A. butzleri*. In contrast to this result, De Smet et al. [41] found that the number of excreting animals and *Aliarcobacter* in the feces did not rise as the animals became older. Giacometti et al. [48] stated that young animals had a much larger proportion of positive samples (27.2% versus 13.15% for adult animals). In another study [13], the number of *A. cryaerophilus* was found to be greater in sheep aged from 1 month to 3 years (11.5%), but it showed a reverse slope for *A. butzleri* (1.6%). The incidence of *A. skirrowii* reached its highest rate between 1 and 3 years (36.1%). 

According to sample type, the frequencies of *Aliarcobacter* species differed considerably in most of the research. Giacometti et al. [48] found that *A. butzleri* was the only species isolated from milk (80%), while *A. cryaerophilus* (12.9%) and *A. skirrowii* (11.2%) were detected as major *Aliarcobacter* species in fecal samples. Celik and Ikiz [13] reported similar results, indicating the sample type was found to be 99% statistically significant (*p* < 0.05), and they also stated that the presence of diarrhea was also found to be statistically significant in the isolation rates (*p* < 0.001) of *A. cryaerophilus* and *A. skirrowii.* In accordance with their results, Hassan [71] stated that cloacal swabs and intestinal samples collected from birds (chickens and turkeys) suffering from enteritis had a greater prevalence rate than samples acquired from healthy birds.

## 2. Prevalence and Antibiotic Resistance of *Aliarcobacter* Species

### 2.1. Aliarcobacter Prevalence Rates in Farm Animal Fecal Content

Due to a lack of specific guidelines, *Aliarcobacter* isolation may not be adequately achieved during regular diagnostic procedures [9], but *Aliarcobacter* has been isolated from the intestines and feces of a variety of domestic animals on several occasions. In a study by Duncan et al. [68], where the researchers worked with dairy cattle and sheep fecal pat, 55.3% and 13.7% of the samples were detected as *Aliarcobacter* spp. in dairy cattle and sheep, respectively. Co-colonization of *Aliarcobacter* species is a widespread situation, and samples containing multiple *Aliarcobacter* species have been found in certain investigations [45,54]. The dominant species isolated from cows was *A. Cryaerophilus*, and co-colonizations, on the other hand, occurred in 26% of the *Aliarcobacter*-excreting animals [21]. The most common species isolated from healthy cattle and sheep was *A. butzleri*, followed by *A. cryaerophilus* and *A. skirrowii* [39,40,72]. Unlike their results, in other investigations, almost all of the species found in the feces were *A. cryaerophilus* and *A. skirrowii* [13,48]. Enteritis, diarrhea, and hemorrhagic colitis have also been associated with *A. butzleri*, *A. skirrowii* and *A. cryaerophilus* [13,73].

### 2.2. Antibiotic Resistance Rates of Aliarcobacter Species in Farm Animals

Increasing antimicrobial drug resistance in food-borne zoonotic pathogens has widespread implications for public health [74]. Phenotypic antimicrobial resistance of *Aliarcobacter* isolates from different sources can be performed with different techniques, including disc diffusion [13,75,76], ETEST^®^ bioMerieux [77,78], agar dilution [79], and broth microdilution [80,81]. Disc diffusion is a culture-based assay that uses antibiotic-containing paper disks to determine antimicrobial susceptibility, and the most popular approaches for determining the minimal concentration of antimicrobial (MIC) drugs that kill or inhibit the growth of microorganisms are agar dilution and broth dilution. The E-test is also used to detect the MIC value of bacteria.

There has been little previous research on the rate of antibiotic resistance genes in *Aliarcobacter* spp. A few studies have demonstrated the antimicrobial resistance mechanism of *Aliarcobacter* with only its chromosomal structure [29,82], but then, as the plasmids that are in charge of antibiotic resistance have been described, the prevalence of the plasmid genes among *Aliarcobacter* spp. has been revealed. Many studies on the antimicrobial susceptibilities of *Aliarcobacter* have been limited mainly to three species: *A. butzleri*, *A. cryaerophilus*, and *A. skirrowii*. Two decades ago, several antimicrobials were recommended for the treatment of *Aliarcobacter* infections. According to Yan et al. [83], cefuroxime (cephalosporin) was the most effective antibiotic used in *Aliarcobacter* medication. Fluoroquinolones have been proposed as an alternative treatment of related intestinal diseases. According to the case, some antibiotics can be used in gastro-intestinal infections, along with quinolones, including tetracycline, macrolide, and b-lactams [84]. Gentamicin and enrofloxacin [54], gentamicin, streptomycin and tetracycline [85], tetracycline, oxytetracycline, erythromycin, ciprofloxacin, kanamycin, amikacin, gentamicin, and enrofloxacin [40] were found as suitable antibiotics that can be used to treat *Aliarcobacter* infections. However, antibiotics that had been chosen for the treatment of *Aliarcobacter* infections started to show resistance against the strains [84]. Some studies have shown that quinolone group antibiotics have started to gain resistance. Quinolone resistance has been connected to the regular use of the drug in animals to prevent disease [86,87]. In *Aliarcobacter* species, a mutation in the quinolone-resistance-determining region of the *gyrA* gene has resulted in significant levels of resistance [16]. 

Tetracyclines are used to a great extent as therapeutics or growth promoters in livestock in China, India, and the United States. That has led to an increase in antibiotic-resistant strains, allergic reactions in humans and animals, and changes in microflora and bacterial populations, but their use as a growth promoter has been prohibited in Europe [88]. The ribosomal protection from tetracycline is given by tetracycline-resistance genes, *tet*(O) and *tet*(W), and Sciortino et al. [89] detected *tet*(O) and *tet*(W) in all resistant *Aliarcobacter* isolates, which was confirmed by disc-diffusion method. *tet*(O) and *tet*(W), present in *A. cryaerophilus*, have been found in high frequency in *A. lanthieri* and *A. faecis* [33]. 

In another study, rectal swabs of cattle and goats were examined, but *Aliarcobacter* species were not found in any of the samples from a goat farm. Resistance against ampicillin, cefotaxime, and ciprofloxacin was detected in *A. butzleri* at 55.6%, 33.4%, and 33.4%, respectively [54]. *A. butzleri* has a large amount of genetic variety and is resistant to several antibiotics, such as amoxycillin+clavulonic acid, nalidixic acid, and ampicillin [80,85].

An increasing number of studies have illustrated that there are differences in susceptibility tests due to the variety of drugs used in animals or the lack of standard susceptibility techniques in *Aliarcobacter* [84,90]. In order to read the results properly, specific breakpoints should be established for defining the resistance in *Aliarcobacter* species. Therefore, to conclude the research, different breakpoint criteria in the Clinical Laboratory Standards Institute (CLSI) have been used. In previous studies, MIC results were compared with breakpoints for *Enterobacteriaceae* or *Staphylococcus* spp., as defined by the CLSI, with breakpoints for *Campylobacter*, or with EUCAST breakpoints for *Enterobacteriaceae*, *Campylobacter*, or non-species-related breakpoints [84,91,92]. Recently, Brückner et al. [9] evaluated MICs with ECOFFs, defined by EUCAST for *C. jejuni*.

Ferreira et al. [87] reviewed the results that were obtained from *Aliarcobacter* antibiotic resistance investigations. The antibiotic resistance variation range was found to be between 4.3 and 14.0% for fluoroquinolones, 0.7 and 39.8% for macrolides, 1.8 and 12.9% for aminoglycosides, and 0.8 and 7.1% for tetracyclines. The high resistance rate reported for *A. butzleri* further shows that this species might behave as a reservoir of genes contributing to antimicrobial resistance transmission through the animal–human–environment interaction, indirectly leading to the failure to treat more severe infections. In addition, *A. butzleri* presented higher resistance rates to penicillin and cephalosporin.

Most *Aliarcobacter* isolates were found to be resistant to β-lactam antibiotics. The most effective compound against *Aliarcobacter* isolates was imipenem [87,93]. The fluoroquinolones, including levofloxacin, marbofloxacin, enrofloxacin, and ciprofloxacin, were detected as effective against *A. butzleri* and *A. cryaerophilus* [93]. According to previous studies conducted on the intestinal content of livestock (Table 2), enrofloxacin, gentamicin, and doxycycline have been understood to have the potential to show efficacy against *Aliarcobacter* strains.

## 3. Genomic Characterization of the Genus *Aliarcobacter*

More than two decades ago, the first entire genome of bacteria was sequenced by Fleischmann et al. [96] and, since then, the sequencing technology and the science of bacteria have developed dramatically. Bacterial diversity, population characteristics, operon structure, mobile genetic elements, and horizontal gene transfer are just a few of the essential issues that genomic data have helped us better comprehend. The accessibility of entire genome sequencing for pathogenic and commensal bacterial species has enabled a more in-depth investigation of their complex relationships with their plant or animal hosts [97].

Currently, a total of 325 genomes of *Aliarcobacter* genus are on the website of the National Center for Biotechnology Information [98]. Currently, 81 belong to *A. butzleri*, 33 belong to *A. cryaerophilus*, and 17 belong to *A. skirrowii*.

Bacterial genomes are now widely sequenced, and data from vast numbers of genomes have a significant influence on our understanding of bacteria. Through genome sequencing, the virulence and the resistance genes can be detected [99]. There are many studies proving this. The antibiotic and metal resistance, along with virulence determinants, were identified by WGS from *A. butzleri* [100]. and *A. cryaerophilus* [101]. The detection of phylogeny, resistance, plasmids, and virulence-associated genes (*ciaB*, *pldA*, *tlyA*, *mviN*, *cadF*, and *cj1349*) of *A. cibarius* and *A. thereius* was carried out by [102]. In another study, the biofilm activity of *Aliarcobacter* isolates on polystyrene, borosilicate, and stainless steel was investigated by biofilm-associated genes (*flaA*, *flaB*, *fliS*, *luxS*, *pta*, *waaF*, and *spoT*), and MLST was applied for the genetic characterization of *Aliarcobacter* strains [103].

Genomic sequencing plays a key role in assessing the risk that *Aliarcobacter* poses for human and animal health. Genomic sequence data may be exchanged to contribute to the understanding of evolution and transmission routes of viruses and bacteria, vaccine development, and diagnostic techniques [104].

## 4. Discussion

Livestock is an integral part of the agricultural production system. It has a key role in the national and global economy [105]. According to the Food and Agriculture Organization of the United States (FAO), livestock contributes 40% of worldwide agricultural production and supports the livelihoods and food and nutrition security of nearly 1.3 billion people. Over 70% of emerging human diseases are caused by animals [106]. Farm animals represent a major source of fecal contamination. Drinking water [107] and food sources [108] that are subjected to feces can lead to disease outbreaks and damage to the economy. The connection between gut microbiota and host health has become apparent over time [109]. Over the last decade, *Aliarcobacter* has been included in numerous studies to explore the gut microbiome. As Collado et al. [110] stated, *Aliarcobacter* may have a fecal origin, as it was detected in the intestinal contents of farm animals, such as chickens, pigs, cattle, sheep, and horses, so that the consumption of farm-animal products may have a potential influence on disease transmission. Although the cases in which *Aliarcobacter* is transmitted to humans from farm animals are rare, previous pathogenesis studies indicate that *Aliarcobacter* has a zoonotic potential [111]. This thought has been backed up by the evidence in a case study where *A. butzleri* was reported to be responsible for a foodborne (roasted chicken) outbreak that occurred at a wedding ceremony in the USA in 2013 [112]. Results obtained in the last decade from different regions showed that the prevalence of *Aliarcobacter* species in farm animal fecal samples ranged from 3 to 100% (Table 1) [45,46]. Generally, *A. butzleri* dominated the gut microbiome of healthy farm animals, as seen in Table 1. There have not been sufficient *Aliarcobacter* investigations in farm animals showing diarrhea symptoms, although many studies were carried out on the stool of humans with gastroenteritis or diarrhea [85,113,114]. However, Celik and Ikiz [13] recently discovered that *A. cryaerophilus* (16%) and *A. skirrowii* (50%) were the key agents in 50 sheep with diarrhea symptoms, whereas *A. butzleri* was found less frequently. Figueras et al. [113] also added that *A. cryaerophilus* was the leading agent of diarrhea. All these results have suggested that *Aliarcobacter* may be a bacteria that should be investigated more in diarrheal diseases in livestock.

There are many factors, including climate, age, and farm conditions, that may play a role in the differences in the prevalence of *Aliarcobacter* species. For instance, Golla et al. [70] explain that there is a direct correlation between age and the prevalence, as adult cattle may have been exposed to different environmental conditions than calves, which may have contributed to the higher *A. butzleri* occurrence. The gut microbiota of younger animals is less likely to be colonized with *A. butzleri* than that of older animals since they have been treated with different nutritional diet plans.

Several antibiotics have been suggested for the treatment of *Aliarcobacter* diseases, but antimicrobial resistance continues to be a major public health issue [115]. Amoxicillin/clavulanic acid, gentamicin, erythromycin, and fluoroquinolones, such as ciprofloxacin and doxycycline, have been reported to be the first-line antibiotics used in the treatment of intestinal infections caused by *Aliarcobacter* spp. [113,116]. As shown in Table 2, the most sensitive drugs were gentamicin, enrofloxacin, oxytetracycline, and doxycycline, which is consistent with findings reported in the last decade. On the contrary, according to many findings, amoxicillin+clavulanic acid, ciprofloxacin, and erythromycin appear to be gaining resistance [13,44,76,94,95]. Ferreira et al. [84] reviewed antibiotic resistance tests that were carried out before 2012. The results of the previous 10 years have been listed in Table 2 of our review, and, as a result, gentamicin and fluoroquinolones (enrofloxacin, oxytetracycline, and doxycycline) are suggested to be used in Aliarcobacter-infected animals. 

When the results are compared, it is clear that the antimicrobial resistance rates in *Aliarcobacter* change over time. For instance, prior to 2012, the majority of *Aliarcobacter* were ciprofloxacin-susceptible [91,117,118], but, recently, some studies conducted with livestock fecal samples have indicated that the rates of resistance to ciprofloxacin have risen (ranging between 22.5% and 44.4%) (Table 2). The resistance range of nalidixic acid changed from 0–64% [117,119] to 25–100% [13,94], and the resistance range of tetracycline expanded from 0–3% [120,121] to 0–100% [40,44]. Moreover, the highest rate of erythromycin resistance was 5% before 2012 [116], whereas it was 100% in 2012 [85]. 

Multi-drug resistance (MDR) has been seen in many farm animals in the past decade. According to Jasim and Al-Abodi [122], there is a significant relationship between some MDR strains and virulence genes in *A. butzleri* and *A. cryaerophilus*. The virulence genes *cadF*, *irgA*, *tylA*, *cdtC*, and *cdtA* were detected in all *A. butzleri* and *A. cryaerophilus* isolates. Furthermore, some *A. butzleri* strains were found to be resistant to tetracycline (72%), amoxicillin (69%), erythromycin (67%), cefoxitin (66%), norfloxacin (43%), and ciprofloxacin (35%), whereas all were found to be susceptible to amikacin, gentamicin, colistin, and fosfomycin. These results highlight the danger of antibiotic resistance in *Aliarcobacter*. This issue can be explained by several molecular mechanisms, including plasmids, transposons, multidrug efflux pumps, and integrons, which have all been implicated in the evolution and spread of multidrug resistance in *Aliarcobacter* [121]. Plasmids are the extrachromosomal element that can transfer genes encoding antimicrobial and heavy-metal resistance, toxins, and virulence phenotypes, and efflux pumps are transport proteins that allow the microorganisms to remove toxic substances from within cells into the surrounding environment [84]. Quinolone remains effective in the treatment of *Aliarcobacter*, but may acquire resistance via efflux and plasmids [123]. As a result, the efficient first-line treatment choices have changed over the last decade, which leaves the breeding and food industries with a number of problems. While aminoglycosides and tetracycline were recommended in 2014 [81] for *Aliarcobater* diseases, now, antibiotic-treated farm animals are becoming less attractive to consumers. This is something that should be borne in mind in future studies. A common source of the antimicrobial resistance of *Aliarcobacter* has been the misuse of antimicrobial drugs, leading to the bacteria acquiring resistance genes from other bacteria and mutations in current resistance genes.The past decade has seen an increase in antimicrobial resistance and, since this can have serious consequences, it is imperative that researchers gain a better understanding of the sources of this issue so that the livestock and human medicine industries can take effective action. 

An important point should also be taken into consideration regarding the differences in the susceptibility of test results. There is still no standard for the *Aliarcobacter* disc-diffusion test, since CLSI has not yet stated any specific breakpoints for evaluating it. This may lead the investigators to misinterpret the results [84]. 

All these studies highlight the need for updated antibiotics for treatment and for further investigation of cases of *Aliarcobacter* in farm animals with diarrhea. More research into the pathogenicity and virulence potential of *Aliarcobacter* species is necessary.

## 5. Conclusions

Over the last decade, *Aliarcobacter* has been included in numerous studies to explore the gut microbiome. Farm animals may be a potential source of *Aliarcobacter*, since high prevalence rates have been detected by many researchers. The fact that *Aliarcobacter* causes diarrhea, enteritidis, and abortion symptoms demonstrates its potential impact on farming and the food industry. Recent research on *Aliarcobacter* distribution and antimicrobial resistance profiles in farm animals has provided a complete understanding of prevention of the disease. However, many factors have been observed to influence the rates. Although the general prevalence was high in autumn and winter, some authors have revealed higher rates of *Aliarcobacter* in summer. In cold and rainy weather, animal welfare and barn hygiene on farms may be poorer. The prevalence of *Aliarcobacter* species might be higher mainly in these seasons, as the animals stay longer in closed barns. For this reason, it is understood that the hygiene of barns should be taken into consideration more since farm animals live in closed areas in winter. The age of farm animals was also not found to be a determinant indicator for the prevalence. Most research has been limited to three species of *Aliarcobacter*—*A. butzleri*, *A. cryaerophilus*, and *A. skirrow*ii. Further studies, therefore, appear necessary to understand the pathogenesis of other *Aliarcobacter* species, but, based on what we know from limited studies, *A. cryaerophilus* and *A. skirrowii* were found frequently in diarrheal cases, indicating that *Aliarcobacter* should be studied more in livestock diarrheal diseases. Despite the fact that *Aliarcobacter* has been found abundant in a small number of studies with farm animals having symptoms of diarrhea, the number of diarrheal cases where *Aliarcobacter* has been investigated is scarce. Researchers need to focus more on this issue. Furthermore, a thorough investigation of the virulence properties of potentially emerging pathogenic bacteria in animals and in foods of animal origin is required for food security. The role of putative virulence determinants in the pathogenicity of *Aliarcobacter* species is still contradictory. Moreover, antibiotic resistance has become a hot topic in animal breeding and the food industry. In the last decade, results showed that *Aliarcobacter*-infected animals could be treated with enrofloxacin, doxycycline, oxytetracycline, and gentamicin. However, these antibiotics are also under threat of acquiring resistance, so a new approach is needed to improve antimicrobial therapeutics.

## Figures and Tables

**Table 1:** Relative (%) prevalence rates of *Aliarcobacter* in intestinal samples of farm animals over the past decade.

Sample	Animal	Sample Size (n)	Prevalence of Aliarcobacter Strains (%)	Clinical Status of Animals	Identification Techniques	References
Fecal samples	Cattle	200	Ab: 58.3	Healthy	PCR	[40]
			Ac: 16.6			
			As: 8.3			
			Ab+Ac: 16.6			
	Sheep	108	Ab: 55	Healthy		
			Ac: 10			
			Ab+Ac: 35			
Chicken swab samples; feces of cattle, sheep and goats	Goats	100	Ab: 18.8	Healthy	PCR and 16S rDNA-RFLP	[44]
			Ac: 31.3			
			As: 12.5			
			A.cl: 12.5			
			An: 6.3			
			Ah: 6.3			
	Sheep	100	Ac: 33.3	Healthy		
			As: 25			
			Ah: 16.7			
			A.cb: 16.7			
			Ab: 8.3			
	Cattle	100	Ab: 38.5	Healthy		
			Ac: 23.1			
			A.cl: 15.4			
			As: 7.7			
			An: 7.7			
	Chickens	100	Ab: 30.3	Healthy		
			Ac: 27.3			
			As: 15.2			
			A.cl: 12.1			
			Acb: 6.1			
Fecal samples	Water buffaloes	30	Ac: 56.6	Healthy and lactation period	PCR and mPCR	[45]
			As: 6.6			
			Ab: 3			
			Ab and Ac: 20			
			As and Ac: 10			
Fecal swabs	Pigs, chickens, turkeys, cattle, sheep, ducks	21	Ab: 16	Healthy	PCR and mPCR	[32]
			Ac: 13			
			As: 12			
Fecal swabs	Cattle	200	Ab: 100	Healthy	PCR and mPCR	[46]
	Sheep	200	Ab: 100			
	Goats	200	Ab: 87.5			
			Ac: 12.5			
Fecal samples	Pigs	135	Ab: 40.7	Healthy	mPCR	[47]
			Ac: 9.6			
			Ab and Ac: 8.9			
	Bovines	75	Ab: 26.7	Healthy		
			Ac: 6.7			
			Ab and Ac: 4			
	Chickens	20	Ab: 10.7	Healthy		
			Ac: 20			
Fecal samples	Sheep	50	Ab: 2	Diarrhea	mPCR	[13]
			Ac:16			
			As: 50			
Fecal samples		50	Ab: 0	Healthy		
			Ac: 0			
			As: 10			
Fecal samples	Cattle	170	Ab: 0.8	Healthy	mPCR and PFGE	[48]
			Ac: 12.9			
			As: 11.2			
Cloacal Swabs and Stool	Ducks, geese, broiler chickens, laying hens	100	Ab: 55	Healthy	PCR and mPCR	[49]
			Ac: 30			
			As: 0			
Fecal samples	Swine	100	Ab: 0	Healthy	mPCR	[50]
			Ac: 12			
Fecal samples	Cattle and calves	792	Ab: 34	Healthy	MLST, WGS, PCR	[51]
			Ac: 49			
			As: 55			
Fecal samples	Cats, cattle, dogs, pigs	197	A.f: 28	Healthy	Real-time (qPCR)	[52]
			A.l: 10			
Fecal samples	Cattle, sheep and broiler chickens	NM	Ab: 67.2	Healthy	PCR	[53]
			Ac: 23.8			
			As: 88.92			
Rectal swabs	Adult cattle (>3 years of age)	110	Ab: 75	Healthy	mPCR	[54]
			As: 12.5			
			Ab + Ac: 12.5			
	Young cattle (<1 year of age)	83	Ab: 0			
			Ab + Ac: 50			
			As + Ac: 25			
			Ab +Ac + As: 25			
	Goats	93	Ab: 0			
			Ac: 0			
			As: 0			

Ab: *A. butzleri*, Ac: *A. cryaerophilus*, As: *A. skirrowii*, Af: *A. faecis*, Al: *A. lanthieri*, Acl: *A. cloaca*, An: *A. nitrofigilis*, Ah: *A. halophilus*, A.cb: *A. Cibarus*, PCR: polymerase chain reaction, mPCR: multiplex polymerase chain reaction, qPCR: quantitative PCR, RFLP: restriction fragment length polymorphism, MLST: multilocus sequence typing, PFGE: pulsed-field gel electrophoresis, WGS: whole genome sequencing, NM: not mentioned.

**Table 2:** *Aliarcobacter* antibiotic resistance rates in percentages in farm animals over the past decade.

Sample	Isolates	AMP	CIP	NAL	GEN	CLOX	TET	ERY	CHL	CTX	ENR	OFX	AMK	OTC	CFZ	GEN + AMX	CEF	DOX	AMC	STM	VAN	CLI	AMX	MET	Reference
**Rectal swab and water swabs**	Ab	56	33	7	4	ND	7	7	7	33	4	ND	ND	ND	ND	ND	ND	ND	ND	ND	ND	ND	ND	ND	[54]
**Fecal samples of cattle and sheep**	Ab (Cattle)	84.1	0	46.1	0	ND	0	0	38.4	ND	7.6	ND	ND	0	92.3	ND	ND	ND	ND	ND	100	84.1	ND	ND	[40]
	Ac (Cattle)	100	0	0	0	ND	0	0	33.3	ND	0	ND	ND	0	100	ND	ND	ND	ND	ND	100	100	ND	ND	
	Ab (Sheep)	88.8	0	55.5	11.1	11.1	0	0	44.4	ND	0	ND	ND	0	100	ND	ND	ND	ND	ND	100	100	ND	ND	
	Ac (Sheep)	50	0	0	0	0	0	0	0	ND	0	ND	ND	0	100	ND	ND	ND	ND	ND	100	100	ND	ND	
**Chicken swab samples; faeces of cattle, sheep, goat, dog and rabbit**	Ab	90	ND	70	ND	100	100	60	ND	ND	5	ND	ND	ND	ND	20	20	10	30	30	25	ND	ND	ND	[44]
	Ac	92	ND	75	ND	100	92	63	ND	ND	8	ND	ND	ND	ND	16	13	21	33	25	36	ND	ND	ND	
	As	100	ND	64	ND	100	100	64	ND	ND	9	ND	ND	ND	ND	18	9	18	18	27	18	ND	ND	ND	
**Faeces and carcass swabs from sheep**	Ab	ND	44.4	100	0	ND	22	0	ND	ND	22	88.9	0	44.5	ND	ND	88.8	11	33	ND	100	ND	22	0	[13]
	Ac	ND	44.4	66	0	ND	0	0	ND	ND	33	100	0	0	ND	ND	88.8	0	44	ND	100	ND	55	88.8	
	As	ND	22.5	25.8	6.5	ND	0	3,2	ND	ND	6.5	29	3	0	ND	ND	90.3	3	32	ND	93.5	ND	35.5	54.8	
**Faeces of pig, poultry, cattle, sheep, and other non-fecal samples**	Ab	ND	0	37.5	18.7	ND	0	50	81.2	ND	ND	ND	ND	ND	ND	ND	ND	ND	ND	ND	100	ND	ND	ND	[94]
	Ac	ND	7.6	30.7	7.6	ND	0	54	76.9	ND	ND	ND	ND	ND	ND	ND	ND	ND	ND	ND	100	ND	ND	ND	
	As	ND	2.4	25	0	ND	0	51	75	ND	ND	ND	ND	ND	ND	ND	ND	ND	ND	ND	100	ND	ND	ND	
**Meat samples of livestock**	Ab	ND	ND	63.4	0	ND	0	49	87.8	ND	ND	ND	ND	ND	ND	ND	ND	ND	ND	ND	ND	70.7	ND	ND	[95]
	Ac	ND	ND	28.6	0	ND	0	71	42.9	ND	ND	ND	ND	ND	ND	ND	ND	ND	ND	ND	ND	71.4	ND	ND	
	As	ND	ND	50	0	ND	0		50	ND	ND	ND	ND	ND	ND	ND	ND	ND	ND	ND	ND	50	ND	ND	
**Broiler cloacal swab**	Ab	0	ND	100	0	ND	0	100	ND	ND	100	ND	ND	ND	ND	ND	ND	ND	0	100	ND	ND	ND	ND	[85]
**Cattle rectal swabs**		71	ND	28.5	0	ND	0	0	ND	ND	0	ND	ND	ND	ND	ND	ND	ND	14.28	100	ND	ND	ND	ND	
**Cloacal swabs from domestic geese**	Ab	66.6	ND	ND	ND	ND	ND	0	66,6	ND	0	0	0	0	100	ND	0	ND	66.6	ND	100	ND	100	ND	[76]
	Ac	85.7	ND	ND	ND	ND	ND	0	0	ND	14.2	0	0	0	100	ND	42.8	ND	28.5	ND	100	ND	14	ND	
	As	71.4	ND	ND	ND	ND	ND	0	28.5	ND	0	0	0	0	100	ND	100	ND	0	ND	100	ND	0	ND	

AMX: amoxycillin, AMP: ampicillin, CLOX: cloxacillin, CHL: chloramphenicol, CTX: cefotaxime, CFZ: cefazolin, CEF: cefalotin, AMC: amoxicillin+clavulanic acid, STM: streptomycin, CLI: clindamycin, CIP: ciprofloxacin, OFX: ofloxacin, ENR: enrofloxacin, NAL: nalidixic acid, AMK: amikacin, DOX: doxycycline, OTC: oxytetracycline, ERY: erythromycin, TET: tetracycline, VAN: vancomycin, MET: methicillin, GEN + AMX: gentamicin+amoxicillin, GEN: gentamicin, Ab: *A. butzleri*, Ac: *A. cryaerophilus*, As: *A. skirrowii*.

## Data Availability

Not applicable.
